# *Vibrio vulnificus* Type 6 Secretion System 1 Contains Anti-Bacterial Properties

**DOI:** 10.1371/journal.pone.0165500

**Published:** 2016-10-31

**Authors:** Selina R. Church, Thomas Lux, Craig Baker-Austin, Sam P. Buddington, Stephen Ll. Michell

**Affiliations:** 1 Biosciences, College of Life and Environmental Sciences, University of Exeter, Exeter, Devon, United Kingdom; 2 Centre for Environment, Fisheries and Aquaculture Sciences, Weymouth Laboratory, Weymouth, Dorset, United Kingdom; Beijing Institute of Microbiology and Epidemiology, CHINA

## Abstract

*Vibrio vulnificus* is a bacterium responsible for severe gastroenteritis, sepsis and wound infections. Gastroenteritis and sepsis are commonly associated with the consumption of raw oysters, whereas wound infection is often associated with the handling of contaminated fish. Although classical virulence factors of this emerging pathogen are well characterised, there remains a paucity of knowledge regarding the general biology of this species. To investigate the presence of previously unreported virulence factors, we applied whole genome sequencing to a panel of ten *V*. *vulnificus* strains with varying virulence potentials. This identified two novel type 6 secretion systems (T6SSs), systems that are known to have a role in bacterial virulence and population dynamics. By utilising a range of molecular techniques and assays we have demonstrated the functionality of one of these T6SSs. Furthermore, we have shown that this system is subject to thermoregulation and is negatively regulated by increasing salinity concentrations. This secretion system was also shown to be involved in the killing of *V*. *vulnificus* strains that did not possess this system and a model is proposed as to how this interaction may contribute to population dynamics within *V*. *vulnificus* strains. In addition to this intra-species killing, this system also contributes to the killing of inter bacterial species and may have a role in the general composition of *Vibrio* species in the environment.

## Introduction

*V*. *vulnificus* is a Gram negative opportunistic pathogen typically isolated from marine and coastal environments [1]. The bacterium is capable of causing an array of disease symptoms such as gastroenteritis, wound infection and primary septicaemia [2]. Wound infection is commonly associated with recreational activities in contaminated water or handling of infected fish and can present with edema, bullae, cellulitis and necrotising fasciitis [2]. In severe cases amputation of limbs may be required. Unlike wound infections which have on average a 25% mortality rate, primary septicaemia, which is often associated with the consumption of contaminated seafood, has a greater than 50% mortality rate rising to 100% without the prompt administration of antibiotic therapy [1]. Currently, in America, the bacterium is the leading cause of seafood related deaths, and due to climate change causing an increase in surface sea temperature, the emergence of this pathogen in colder regions such as the Baltic Sea are becoming increasingly common [3].

Although this pathogen is known to be highly pathogenic, the number of severe human infections reported each year is extremely low given the virulence potential and environmental prevalence of this bacterium [4]. This has led researchers to believe that not all strains are equal in virulence, with some strains better adapted to causing human disease than others [5]. However, a genetic basis for this difference in virulence potential is yet to be established. Furthermore, there is limited knowledge surrounding the general biology of *V*. *vulnificus* and to date only two secretion systems have been characterised in this bacterium, the type 1 secretion system (T1SS) and the type 2 secretion system (T2SS) [6]. The former is known to secrete the well-studied toxin RtxA which has been shown to be required for both gut pathogenesis and dissemination throughout the body [7], whereas the latter is required for the secretion of the haemolysin, VvhA [6], a toxin which is also required for gut pathogenesis [7]. To investigate the presence of previously unreported virulence factors in *V*. *vulnificus*, we performed whole genome sequencing which led us to identify previously unreported T6SSs.

The T6SS is found in approximately 25% of all sequenced Gram negative bacterial genomes, and is made up of 13 conserved proteins [8]. These 13 proteins are hypothesised to form a macromolecular structure similar to the contractile tail of bacteriophage [9]. The needle like structure of the T6SS is composed of Hcp, VrgG and PAAR proteins [10], with ATPase activity being provided by ClpV and the core scaffolding protein, IcmF [11, 12]. Unlike previously identified secretion systems, which usually export anti-eukaryotic effectors, the T6SS has dual functions, secreting both anti-eukaryotic and anti-prokaryotic effectors [13]. The latter function in particular has captured the attention of many researchers, as it poses the potential for development of novel antimicrobials [14]. In addition to secreting anti-bacterial toxins, it has also been shown that anti-bacterial T6SS encoding bacteria also harbour immunity proteins. These immunity proteins are able to neutralise the deleterious effects of toxins to prevent self-intoxication and provide immunity between T6SS harbouring sister cells [14, 15].

To study the role of the T6SS in affecting the population dynamics of *Vibrio* spp. this study employed co-culturing assays. These assays demonstrated that the T6SS present in *V*. *vulnificus* can play an important role in bacterial population composition through anti-bacterial activity. Furthermore, we present a hypothesis describing how the T6SS may provide an explanation for the limited number of serious human infections attributed to *V*. *vulnificus* given the high abundance of this bacterium in the environment.

## Results

### *V*. *vulnificus* strains vary in their T6SS genetic architecture

**Fig 1:**
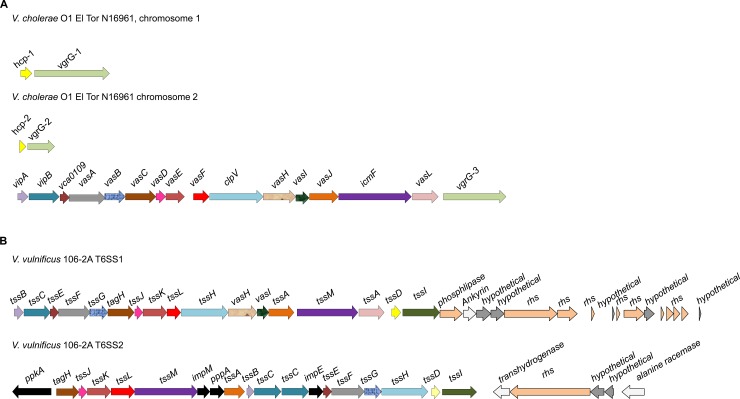
T6SSs of *Vibrio vulnificus*. (A) Schematic diagram showing the genetic organisation of T6SS of *V*. *cholerae*. (B) Schematic representation of the genetic organisation of T6SSs from *V*. *vulnificus*. Accessory proteins present in T6SS2 and absent from T6SS1 are shown in black. Homologous genes between the two species have been colour matched to represent their similarity.

In an approach to identify genetic differences between *V*. *vulnificus* isolates that may account for species specific phenotypes, whole genome sequencing was applied to a panel of ten strains of varying virulence potential and isolation source (Table 1) [5]. Analysis of this data identified a novel T6SS, herein referred to as T6SS2, which was present in all ten sequenced isolates (S1 Fig). Furthermore, an additional T6SS, present in three out of the ten isolates, T6SS1, was also identified (S2 Fig). This T6SS1 exhibited synteny with the previously characterised T6SS from *V*. *cholerae* [16] having 76% similarity over 19.2 kb. However, several differences between T6SS1 of *V*. *vulnificus* and the T6SS of *V*. *cholerae* exist and are depicted in Fig 1. For example, *V*. *cholerae* (Fig 1A) contains two *hcp* genes, which are distributed across chromosomes 1 and 2 whereas the sole *hcp* gene of *V*. *vulnificus* T6SS1 is located within the T6SS operon on chromosome 2 (Fig 1B). Both T6SS1 and T6SS2 of *V*. *vulnificus* contain the 13 core genes required for a functional T6SS (Fig 1B) [8]. However, T6SS2 contains several accessory genes, encoding for a phosphatase and kinase, which are absent from T6SS1. Bioinformatic investigation of three *V*. *vulnificus* reference genomes, YJ016, MO6-24/O and CMCP6 revealed that all three genomes did not contain T6SS1 but all had T6SS2, although the latter were not annotated as such in their Genbank depositions (S1 Fig).

**Table 1:** Bacterial strains.

Bacterial strains	Description	Virulence Grouping ^a^	Source
*V*. *vulnificus* 106-2A	Wild-type strain, environmental isolate, T6SS1+ T6SS2+	3	CEFAS
*V*. *vulnificus* 99–743	Environmental isolate, T6SS1- T6SS2+	3	CEFAS
*V*. *vulnificus* MO6-24/O	Clinical isolate, T6SS1- T6SS2+	4	CEFAS
*V*. *vulnificus* 99–796	Environmental isolate, T6SS1+ T6SS2+	4	CEFAS
*V*. *vulnificus* ORL-1506	Clinical isolate, T6SS1- T6SS2+	5	CEFAS
*V*. *vulnificus* NSV-5830	Clinical isolate, T6SS1- T6SS2+	5	CEFAS
*V*. *vulnificus* DAL-79087	Clinical isolate, T6SS1- T6SS2+	3	CEFAS
*V*. *vulnificus* DAL-79040	Clinical isolate, T6SS1- T6SS2+	2	CEFAS
*V*. *vulnificus* S3-16	Environmental isolate, T6SS1+ T6SS2+	1	CEFAS
*V*. *vulnificus* S2-22	Environmental isolate, T6SS1- T6SS2+	3	CEFAS
*V*. *vulnificus* ATL-9824	Clinical isolate, T6SS1- T6SS2+	2	CEFAS
*V*. *fluvialis* NCTC 11327	Clinical isolate	N/A	CEFAS
*V*. *vulnificus* SRC1	106-2A Δ*icmF1* in-frame, T6SS1- T6SS2+	N/A	This study
*V*. *vulnificus* SRC2	106-2A Δ*icmF2* in-frame, T6SS1+ T6SS2-	N/A	This study
*V*. *vulnificus* 106-2A pSCrhaB3	Wild-type strain containing pSCrhaB3 (Tp^r^)	N/A	This study
*V*. *vulnificus* 106-2A:*icmF1* pSCrhaB3	106-2A Δ*icmF1* containing pSCrhaB3 (Tp^r^)	N/A	This study
*V*. *vulnificus* 106-2A:*icmF2* pSCrhaB3	106-2A Δ*icmF2* containing pSCrhaB3 (Tp^r^)	N/A	This study
*V*. *vulnificus* 99–743 pBHR4-groS-RFP	*V*. *vulnificus* 99–743 containing pBHR4-groS-RFP (Cm^r^)	N/A	This study
*V*. *fluvialis* NCTC 11327 pBHR-4groS-RFP	*V*. *fluvialis* NCTC 11327 containing pBHR4-groS-RFP (Cm^r^)	N/A	This study
*V*. *vulnificus* MO6-24/O pBHR-4groS-RFP	*V*. *vulnificus* MO6-24/O containing pBHR4-groS-RFP (Cm^r^)	N/A	This study
*V*. *cholerae* V52	Wild-type strain (Sm^r^)	N/A	Dr. S. Pukatizki
*Escherichia coli* DH5α	Cloning host	N/A	Lab stock
*Escherichia coli* S17 λpir	Cloning host and donor strain	N/A	Lab Stock
*Escherichia coli* TOP10	Cloning host	N/A	Invitrogen

^a^ Virulence grouping was determined from the *in vivo* study performed by Thiaville *et al*., 2011, virulence increases with numerical value.

### *V*. *vulnificus* T6SS1 secretes Hcp-1 in a temperature and salinity dependent manner

**Fig 2:**
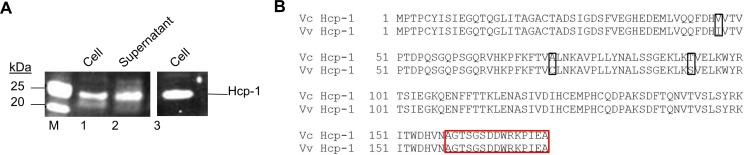
T6SS1 of *V*. *vulnificus* is functional. (A) Western blot employing an antibody against Hcp-1 from *V*. *cholerae* demonstrating the presence of Hcp-1 in the culture filtrate and cell pellet of *V*. *vulnificus* 106-2A (lane 1 and 2). Lane 3, protein extract from *V*. *cholerae* V52 used as a positive control to confirm the specificity of the antibody (14 μg of protein per lane). (B) Amino acid alignment of Hcp-1 from *V*. *cholerae* and *V*. *vulnificus*. Amino acid differences are boxed in black and epitope to which the anti-Hcp-1 antibody was raised is highlighted in red.

As T6SS1 of *V*. *vulnificus* exhibits synteny with the previously described T6SS of *V*. *cholerae*, we first sought to demonstrate that T6SS1 was functional in *V*. *vulnificus*. This was assessed by confirming secretion of Hcp-1, a prototypical T6SS secreted component. Western blot analysis using an antibody against the Hcp peptide sequence; AGTSGSDDWRKPIEA [17] confirmed that Hcp-1 was secreted into the culture filtrate (Fig 2A). We were able to use the anti- Hcp-1 antibody raised against Hcp-1 from *V*. *cholerae* due to the high level of homology between *V*. *cholerae* and *V*. *vulnificus* (Fig 2B). Previous characterisation of T6SSs from *V*. *cholerae* and *V*. *parahaemolyticus* has demonstrated that salinity and temperature can affect activation of these systems [18, 19]. We therefore investigated the effect of temperature and salinity on T6SS1 of *V*. *vulnificus* in order to determine whether it was similarly regulated.

**Fig 3:**
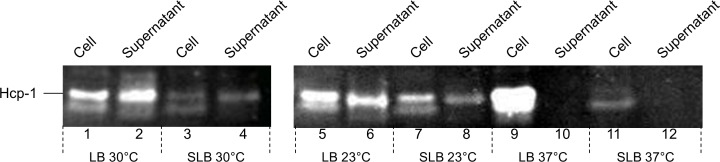
Hcp-1 expression from T6SS1 of *V*. *vulnificus* 106-2A is dependent upon temperature and salinity. Western blot of cell lysates and supernatants from *V*. *vulnificus* 106-2A cultures grown at 23°C, 30°C and 37°C, using anti-Hcp-1 antibody. Cultures were grown in LB or LB supplemented with 3% NaCl w/v (SLB) (14 μg of protein per lane).

The Western blot using the anti- Hcp-1 antibody shown in Fig 3 demonstrates that Hcp-1 is secreted at 23°C and 30°C (lanes 2 and 6), temperatures similar to those seen in marine environments but not at 37°C (lane 10). When *V*. *vulnificus* was cultured at these temperatures in the presence of 3% w/v NaCl, a similar pattern of secretion was observed but at lower levels (cf; lanes 4, 8 and 12). Qualitative analysis of the cell lysates of these experiments demonstrated that Hcp-1 was expressed under all conditions but with secretion by the T6SS1 reduced by high temperature and increased salinity.

### *V*. *vulnificus* utilises T6SS1 in intra-species killing

**Fig 4:**
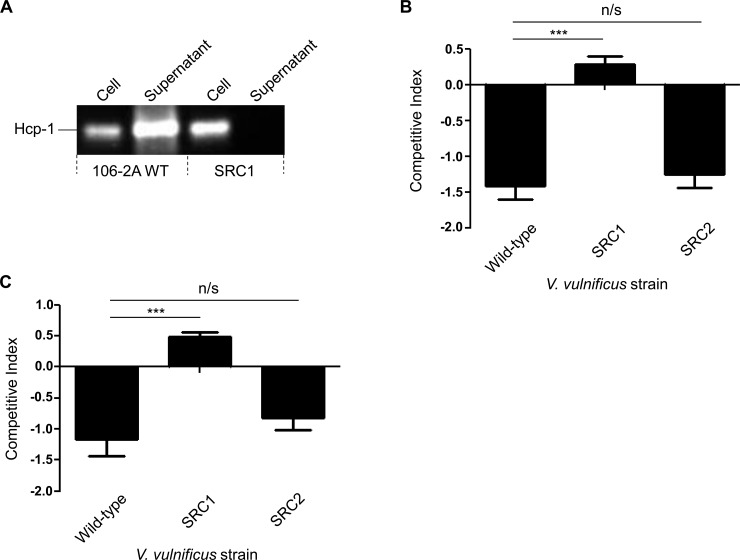
**T6SS1 targets T6SS1 deficient strains of V. vulnificus** (A) Western blot employing an antibody against Hcp-1 demonstrating the presence of Hcp-1 in the supernatant and cell pellet of *V*. *vulnificus* 106-2A wild-type. Whereas, Hcp-1 is only detected in the cell pellet of the *icmF1* mutant, SRC1 and not in the supernatant. (B) Competitive index of *V*. *vulnificus* 99–743 (prey) following co-culture for two hours with *V*. *vulnificus* 106-2A (attacker) demonstrating an increase in the survival of the naturally occurring T6SS1 negative prey strain, 99–743 when co-cultured with SRC1 compared to 106-2A wild-type. (C) Competitive index of *V*. *vulnificus* MO6-24/O (prey) following co-culture for two hours with *V*. *vulnificus* 106-2A (attacker). Competitive index was calculated using the following equation: (input attacker/input prey)/(output attacker/output prey). Statistical analysis was performed using the Wilcoxon signed-rank test on log-transformed data. *** = p < 0.0005.

The T6SS of *V*. *parahaemolyticus* is active against other bacteria at marine like temperatures [18]. Therefore, we hypothesised that T6SS1 of *V*. *vulnificus* may be similarly involved in bacterial killing as this system is also functional under the same conditions observed for *V*. *parahaemolyticus* [18]. To test this hypothesis we first sought to determine whether a *V*. *vulnificus* T6SS1 positive strain could kill a *V*. *vulnificus* T6SS1 negative strain, based on the rationale that the latter strain would be deficient in immunity proteins to T6SS1 effectors. This was investigated by performing co-culture assays at 30°C using a naturally occurring T6SS1 negative *V*. *vulnificus* environmental strain, (99–743) as prey and, a T6SS1 positive environmental strain, (106-2A) as the attacker. These strains were chosen as 106-2A contains both T6SS1 and T6SS2, whereas 99–743 is a naturally occurring T6SS1 mutant. To test whether T6SS1 conferred a competitive advantage in 106-2A, an in-frame deletion of *icmF1*, (a gene encoding a core scaffolding protein of the T6SS) was created in strain 106-2A, resulting in strain SRC1. This mutation was confirmed by whole genome sequencing (S3 Fig). To confirm that this mutation resulted in an inactive T6SS1, Western blot analysis was performed to observe secretion of Hcp-1. As can be seen in Fig 4A, SRC1 was unable to secrete Hcp to the culture filtrate at 30°C when compared to the wild type strain. Furthermore, for enumeration purposes the competitive assay was performed using the prey strain, 99–743 which has been engineered to be chloramphenicol resistant and the attacker strains, 106-2A, SRC1 to be trimethoprim resistant. In addition to SRC1, an in-frame *icmF2* mutant was generated in T6SS2 (SRC2) to determine whether T6SS2 played a role in bacterial targeting.

The competitive assays demonstrated that strain 106-2A could indeed target 99–743 at 30°C, resulting in a significant reduction in numbers of the prey strain (Fig 4B). In contrast to the observed inhibition of growth of the prey strain 99–743 when co-cultured with wild-type 106-2A, co-culture of 99–743 with SRC1 resulted in a significant increase in the competitive index of the prey strain (Fig 4B). Furthermore, we demonstrated that T6SS2 of 106-2A did not inhibit 99–743 as evidenced by co-culture with an *icmF2* in-frame deletion mutant of 106-2A (SRC2). This latter experiment resulted in a phenotype comparable to that of the wild-type strain, Fig 4B.

As the prey strain 99–743 is an environmental isolate, we sought to determine whether T6SS1 of 106-2A could target a hyper-virulent clinical isolate. In order to test this hypothesis the hyper-virulent reference strain, MO6-24/O which was isolated from a patient with septicaemia [20] was selected as a prey strain for use in the co-culture assays. These co-culture assays demonstrated that as was the case for 99–743, the hyper-virulent clinical strain MO6-24/O was inhibited by the environmental strain 106-2A in a T6SS1 dependent manner, as evidenced by a lack of killing by SRC1 (the *icmF1* mutant strain) Fig 4C.

### *V*. *vulnificus* utilises T6SS1 in inter-species killing

T6SSs of several bacteria have been shown to play a role in effecting microbial population dynamics through inter-species killing [21]. In addition, it is known that several pathogenic *Vibrio* species exist alongside *V*. *vulnificus* within marine environments and bivalve molluscs [22]. We hypothesised that the T6SS1 of *V*. *vulnificus* may, in addition to the intra-species killing demonstrated above, play a role in effecting its proximal marine microflora through inter-species killing by T6SS1. We selected *Vibrio fluvialis* as a model bacterium to test this hypothesis as *V*. *fluvialis*, along with *V*. *vulnificus*, is known to be a shellfish-associated bacterium [23]. Furthermore, these strains were employed as post co- culture enumeration was performed on TCBS agar, on which *V*. *vulnificus* 106-2A forms green colonies and *V*. *fluvialis* forms yellow colonies. TCBS enumeration plates were incubated at 37°C, at which temperature T6SS1 did not secrete Hcp-1 (Fig 3, lane 10), excluding the possibility that killing due to T6SS1 was occurring on the enumeration plates.

Unlike the previous intra-species assays, the competitive index could not be calculated for either *V*. *fluvialis* or *V*. *vulnificus*. This is due to the complete removal of *V*. *fluvialis* and *V*. *vulnificus* in several of the assays resulting in an enumeration count of zero. Therefore, the number of recovered cells following co-culture is presented in place of the competitive index value.

**Fig 5:**
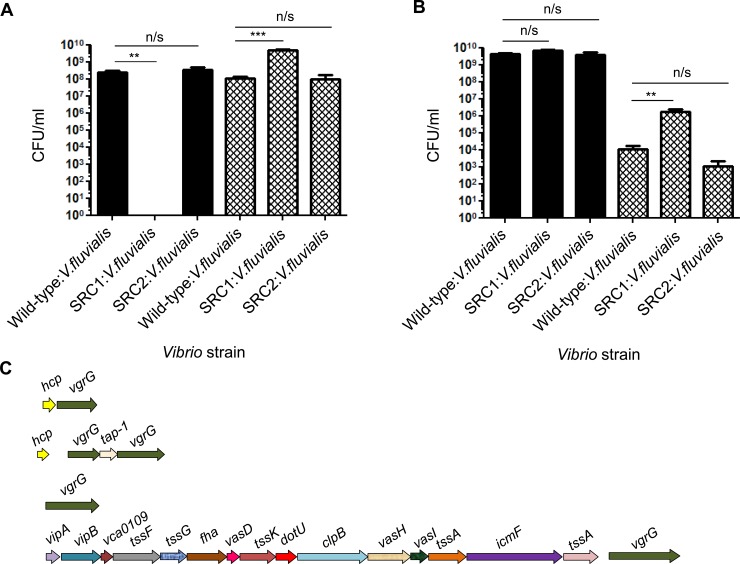
*V*. *vulnificus* utilises T6SS1 for inter-species targeting at 30°C. (A) The CFU/ml of recovered bacteria was recorded following a co-culture T6SS killing assay at 30°C for 5 hours when T6SS1 is active in the attacker strain. Attacker strain *V*. *vulnificus* 106-2A (solid bars) Prey strain *V*. *fluvialis* NCTC11327 (hatched bars). Co culture enumeration was performed at 37°C on TCBS where the T6SS is not active in the attacker strain. Statistical analysis was performed using a 1-way paired Student’s t-test, ** = p < 0.005. (B) Recovered CFU/ml of bacteria following co-culture at 30°C and enumeration at 37°C using antibiotic resistant strains on antibiotic plates. Hatched bars represent the prey strain, *V*. *fluvialis* NCTC11327. Solid bars represent *V*.*vulnificus* attacker strains. Statistics were performed using the 2-way unpaired Student’s t-test *** = p < 0.005. (C) Schematic diagram of the *V*. *fluvialis* T6SS gene cluster and distally encoded T6SS associated genes.

In co-culture assays where *V*. *fluvialis* NCTC 11327 was used as a prey strain, and the T6SS1 mutant of *V*. *vulnificus* (SRC1) as the attacker, it was observed that *V*. *fluvialis* exhibited a significant increase in growth compared to when co-cultured with wild-type *V*. *vulnificus* at 30°C (Fig 5A, hatched bars). This suggests that the T6SS1 of *V*. *vulnificus* is involved in the inter-species killing of *V*. *fluvialus*. Furthermore, this observed phenotype is unlikely to be attributable to the T6SS2 of *V*. *vulnificus*, as there was no significant difference in the survival of *V*. *fluvialis* when co-cultured with the T6SS2 mutant (SRC2) compared to when co-cultured with wild-type *V*. *vulnificus* (Fig 5A, hatched bars).

However, it was also observed that when *V*. *fluvialis* was co-cultured with the T6SS1 mutant of *V*. *vulnificus* (SRC1), this latter strain appeared to become a prey strain for *V*. *fluvialis* as evidenced by a complete lack of growth of SRC1 (Fig 5A, solid black bars). Therefore, we reasoned that this observed inhibition of SRC1 growth may be attributable to a T6SS present in *V*. *fluvialis* which is active during enumeration at 37°C. To test this hypothesis, we generated antibiotic resistant *V*. *vulnificus* and *V*. *fluvialis* strains allowing for the prey and attacker strains to be enumerated on separate antibiotic plates. This approach removed the ability for intra-strain targeting to occur during enumeration at 37°C. Enumeration of a subsequent co-culture assay on antibiotic containing enumeration plates revealed that there was no inhibition of SRC1 (Fig 5B, solid black bar), suggesting that *V*. *fluvialis* may indeed be targeting SRC1 at 37°C on TCBS agar. This experiment also revealed that during the co-culture at 30°C, *V*. *vulnificus* can target *V*. *fluvialis* in a T6SS1 dependent manner as evidenced by an increase in growth of *V*. *fluvialis* when co-cultured with the SRC1 compared to wild-type and SRC2 (Fig 5B, hatched bar).

### Identification of a T6SS in *V*. *fluvialis* NCTC 11327

To determine whether *V*. *fluvialis* contained a T6SS, which may be contributing to the observed targeting of SRC1, whole genome sequencing of *V*. *fluvialis* NCTC 11327 was employed. This approach, coupled with genome annotation, did identify a putative T6SS in *V*. *fluvialis*, Fig 5C. This system contains all of the 13 conserved genes required for a functional T6SS and is similar in gene organisation to T6SS1 of *V*. *vulnificus*. However, unlike *V*. *vulnificus*, *V*. *fluvialis* encodes two *hcp* genes, which are located distally from the large T6SS operon. This localisation of *hcp* genes is similar to that seen in *V*. *cholerae* [24]. Furthermore, *V*. *fluvialis* also possesses distally encoded *vgrG* genes, as well as a homologue of the recently identified T6SS adapter protein-1 (*tap-1*) (Fig 5C) [25].

### The T6SSs in *V*. *vulnificus* 106-2A are not virulent towards *Galleria mellonella*

**Fig 6:**
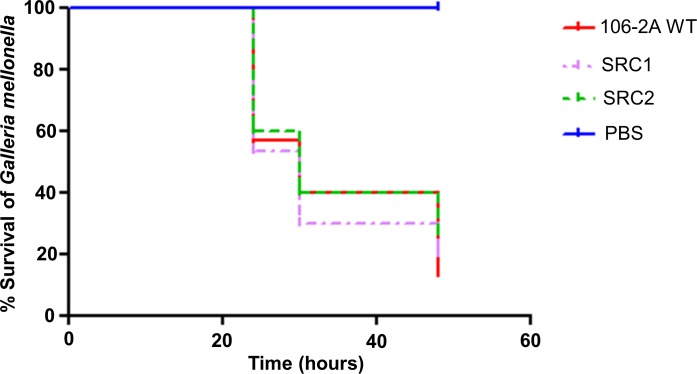
The T6SSs of *V*. *vulnificus* do not contribute towards virulence in the infection model, *Galleria mellonella*. *V*. *vulnificus* is virulent in the infection model *G*. *mellonella*, however neither the *icmF* T6SS1 mutant, SRC1 nor the *icmF* T6SS2 mutant, SRC2 are attenuated in *G*. *mellonella*. Phosphate buffered saline (PBS) was used as a negative control demonstrating 100% survival. Experiments were performed three times in triplicate where n = 10 and statistics performed using GraphPad Prism version 5 using the Log-rank (Mantel-Cox) test.

To further support our hypothesis that the T6SS1 of *V*. *vulnificus* is predominantly involved in anti-bacterial killing rather than anti-eukaryotic targeting, we investigated the role of T6SS1 in a *Galleria melonella* infection model. Groups of 10 larvae were injected with either, *V*. *vulnificus* 106-2A wild-type, SRC1 or SRC2 at 10^5^ cells in the upper most pro-leg and monitored for signs of disease. The results however demonstrated that both SRC1 and SRC2 were as virulent compared to the wild-type strain, Fig 6, indicating that neither T6SS1 nor T6SS2 is required by *V*. *vulnificus* for virulence in this infection model, further supporting our hypothesis that the T6SS1 of *V*. *vulnificus* is involved in modulating the composition of environmental bacterial populations.

## Discussion

Although T6SSs have been characterised in many *Vibrio* species, this is the first report, to our knowledge, of a functional T6SS in *V*. *vulnificus*. Our study has shown a variation in the number of T6SSs present in ten different *V*. *vulnificus* isolates, with some having two T6SSs and others only one. This in contrast to *V*. *cholerae*, where numerous strains have been shown to possess just one conserved T6SS [26]. All *V*. *vulnificus* strains studied contained the T6SS, T6SS2 with a subset of strains containing an additional T6SS, T6SS1. Bioinformatic analysis of both T6SSs of *V*. *vulnificus* demonstrated that these systems contained the full repertoire of genes required for a functional secretion system [8]. *V*. *vulnificus* T6SS2 contained accessory genes encoding for proteins such as a phosphatase and kinase, which have previously been shown to be required for T6SS regulation in *P*. *aeruginosa* [27]. Although the accessory proteins observed in T6SS2 were not observed in T6SS1, T6SS1 does contain *vasH*. This gene encodes for an alternative sigma^54^ factor, which has previously been shown to be involved in initiation of transcription of the T6SS in *V*. *cholerae* [28, 29] and as such may have a regulatory role in the transcription of T6SS1 in *V*. *vulnificus*.

T6SS1 of *V*. *vulnificus* exhibits considerable overall synteny with the T6SS of *V*. *cholerae* [30]. However, the most notable differences between the T6SSs of these bacteria are predominantly associated with the *hcp* and *vgrG* genes. For example, *V*. *cholerae* contains two *hcp* and three *vgrG* genes, in contrast to the apparent presence of only one copy of each in the *V*. *vulnificus* T6SSs. VgrGs play a structural role in the formation of the T6SS, however several *vgrGs* have also been shown to encode for effector functions, such as those identified in *V*. *cholerae*, and as such are denoted as evolved VgrGs [17, 24]. Evolved *vgrGs* are distinguishable from structural *vgrGs* based on the presence of a C-terminal extension. The *vgrG* genes in T6SS1 and T6SS2 of *V*. *vulnificus* appear not to be evolved *vgrG* genes due to the lack of a C-terminal extension.

In this paper we show for the first time that T6SS1 of *V*. *vulnificus* is functional. Expression and secretion of Hcp-1 was shown to be affected by both temperature and salinity as has been observed previously in both *V*. *cholerae* and *V*. *parahaemolyticus* [18, 19]. Our initial findings demonstrated that the temperature regulation of *V*. *vulnificus* Hcp-1 was identical to those of Hcp-2 in *V*. *parahaemolyticus* [18]. In contrast to this temperature dependent similarity, salinity appeared to have a differential effect on secretion of *V*. *vulnificus* Hcp-1 relative to *V*. *parahaemolyticus* Hcp-2 [18]. Differential regulation of T6SSs in other *Vibrio* species is not uncommon, as *V*. *cholerae* V52 Hcp has been shown to be secreted at 37°C but Hcp of the O1 *V*. *cholerae* strain, A1552, is secreted at only 23°C [19, 30]. Our observation that Hcp-1 is not secreted at 37°C suggests that the function of *V*. *vulnificus* T6SS1 is unlikely to be associated with an anti-eukaryotic phenotype. A hypothesis supported by our observation that SRC1 displays the same virulence in a *G*. *mellonella* model of infection to that of the wild-type.

Based on T6SS1’s temperature regulation and similarity with the anti-bacterial T6SS of *V*. *cholerae*, [21] we hypothesised that *V*. *vulnificus* T6SS1 may exhibit anti-bacterial properties. Furthermore, our observation that only some strains of *V*. *vulnificus* contain this additional T6SS1 led us to propose that this system may convey a competitive advantage. Through the use of co-culturing assays this hypothesis was supported by the observation that T6SS1 positive strains do have a competitive advantage over those that are T6SS1 negative. Furthermore, mutagenesis studies demonstrated that this phenotype was attributed to T6SS1 and not T6SS2.

Given the observed antagonistic role of *V*. *vulnificus* T6SS1, it is tempting to speculate on this system’s role in the natural environment, and potentially more widely in terms of clinical impact. Taken together, we hypothesise that the limited number of serious *V*. *vulnificus* human infections compared with the high abundance of this pathogen [4] may be attributable to T6SS1 dependent killing of certain hyper virulent *V*. *vulnificus* strains. This hypothesis is based on data in this study which has shown that a lesser virulent *V*. *vulnificus* strain can target a hyper virulent strain in a T6SS1 dependent manner, leading us to suggest that the same scenario may also occur naturally in the environment. This proposed scenario would potentially lead to a decrease in the number of hyper virulent strains in the environment, which may lessen the chances of humans being exposed to hyper virulent strains, and as such only a limited number of serious cases reported each year. This scenario is appealing as our study identified a predominance of T6SS1 among lesser virulent environmental strains [5]. In accordance with our hypothesis, other researchers have also suggested that the main role of the T6SS is to attack competing cells and enhance the fitness of the secreting cell in poly-microbial communities [14, 31]. However, it should be noted that although this study identified a predominance of T6SS1 among lesser virulent strains, T6SS1 was also identified in a hyper virulent environmental strain, 99–796. Furthermore, given the increase in knowledge regarding different effector/immunity proteins in *V*. *cholerae* allowing for differing strains of *V*. *cholerae* to outcompete each other based on their effector repertoire [26] this hypothesis may be too simplistic. Initial *in silico* investigations into the *V*. *vulnificus* T6SS1 and T6SS2 effectors has demonstrated that *V*. *vulnificus* contains mainly rhs effectors with DNase activity. However, to fully answer our proposed hypothesis the identification of *V*. *vulnificus* T6SS1 and T6SS2 effectors and immunity proteins needs to be elucidated, a study which our laboratory is currently undertaking.

As well as intra-species targeting, bacterial species such as *V*. *cholerae* and *S*. *marcescens* utilise their T6SS for inter-species targeting [21, 32]. Therefore, we sought to determine whether *V*. *vulnificus* T6SS1 could target other bacterial species. To test this hypothesis, co-culture assays were performed using the prey strain, *V*. *fluvialis* NCTC 11327. Both the anti-bacterial properties of T6SS1 and T6SS2 were investigated as it was hypothesised that T6SS1 may be specific to intra-species targeting and T6SS2 inter-species targeting. However, the results demonstrated that T6SS2 played no role in inter-species targeting. The finding that T6SS2 plays no role in anti-bacterial killing is not unusual as bacteria containing multiple T6SSs can often utilise these system for distinct roles. For example, although *B*. *thailandensis* contains a total of five T6SS, only T6SS-1 has been shown to be anti-bacterial, while T6SS-5 has been shown to possess an anti-eukaryotic phenotype [33]. Although our studies did not demonstrate any role for *V*. *vulnificus* T6SS2, it is likely that this system is tightly regulated. This hypothesis is based on the finding that T6SS2 contains the accessory proteins, phosphatase and kinases, two proteins which have been shown to be involved with tight regulation of the T6SS, HSI-I in *P*. *aeruginosa* [27].

During co-enumeration on TCBS agar, the *V*. *vulnificus* T6SS1 mutant, SRC1 was unable to survive in the presence of *V*. *fluvialis* NCTC 11327 at 37°C. Suggesting that *V*. *vulnificus* requires T6SS1 for survival in mixed bacterial populations. Based on these findings, WGS and annotation was performed to determine whether *V*. *fluvialis* NCTC 11327 contained a T6SS which may account for the antagonistic manner of this strain. These investigations identified the presence of a T6SS locus in *V*. *fluvialis* NCTC 11327. Although the functionality of this system was not investigated, the T6SS locus contains all of the 13 genes required for a functional T6SS [8] and is similar in gene organisation to the T6SS of *V*. *cholerae* V52 [24]. Similarly, a recent bioinformatic study using the *V*. *fluvialis* strain, 85003 also identified the presence of a T6SS cluster, suggesting that the T6SS is common among *V*. *fluvialis* strains [34]. The widespread identification of the T6SS in the *Vibrio* family is not surprising as many *Vibrio* spp inhabit similar niches, therefore containing a mechanism such as the T6SS which can displace competing bacteria when resources such as space and nutrients are limited would be beneficial [14].

In addition to environmental attacks, a recent publication demonstrated the antagonistic behavior of the T6SS *in vivo* [31]. The authors extrapolated this information to suggest the pivotal role played by the T6SS in affecting the composition of many poly-microbial communities and the effects of this system on health and disease outcomes [31]. Similarly, we hypothesise that the T6SS may play an important role in determining the severity of *V*. *vulnificus* disease. For example, *V*. *vulnificus* and *V*. *fluvialis* share the oyster as a common niche and human infection route [4, 23] and in this study we have demonstrated that at 37°C in the absence of T6SS1, *V*. *fluvialis* can target *V*. *vulnificus*. Furthermore, we have identified that the majority of *V*. *vulnificus* strains are T6SS1 negative. Therefore, we propose that upon human ingestion of an oyster containing *V*. *fluvialis* and T6SS1 negative *V*. *vulnificus* cells, *V*. *fluvialis* may target and remove *V*. *vulnificus*. In comparison to *V*. *vulnificus* which can exhibit varying degrees of pathogenicity [5], *V*. *fluvilais* is commonly associated with mild gastroenteritis [23]. Therefore, we speculate that in this instance, *V*. *fluvialis* could acts as a “probiotic”, by targeting and removing hyper virulent *V*. *vulnificus* cells, leaving only the lesser virulent *V*. *fluvialis* for the host’s immune system to eliminate. Based on this hypothesis, it is tempting to conclude that only a limited number of serious *V*. *vulnificus* infections would be reported each year. Further work is required to understand whether the removal of *V*. *vulnificus* by *V*. *fluvialis* is due to the *V*. *fluvialis* T6SS or whether there are potentially other diffusible anti-microbial molecules secreted by *V*. *fluvilais*. This is based on the finding that *V*. *fluvilais* was able to target *V*. *vulnificus* on enumeration plates where colonies were sufficiently diluted to not be in contact. It is highly likely that *V*. *fluvialis* may secrete diffusible anti-microbial molecules as other *Vibrios* have previously been shown to secrete anti-microbial molecules other than those associated with the T6SS [35].

In conclusion, this study has identified for the first time two T6SSs in *V*. *vulnificus*. Furthermore, we have identified the conditions under which T6SS1 is active, and demonstrated that T6SS1 contains anti-bacterial properties. These findings have been used to hypothesise how *V*. *vulnificus* T6SS1 may be used to shape *Vibrio* microbial populations and how inter-bacterial interactions may explain the limited number of serious human infections attributed to *V*. *vulnificus* given the natural prevalence and virulence potential of this bacterium.

## Materials and Methods

### Bacterial strains, plasmids and culture conditions

*V*. *vulnificus* strains and *V*. *fluvialis* NCTC 11327, were routinely cultured at 30°C and *V*. *choerlae* V52 at 37°C in Luria-Bertani (LB) broth (Oxoid) unless otherwise stated. Where appropriate, antibiotics (Sigma) were added at the following concentrations; ampicillin (Amp) 100 μg/ml, Kanamycin (Km) 50 μg/ml, chloramphenicol (Cm) 35 μg/ml for *Escherichia coli* and 10 μg/ml for *Vibrio vulnificus*, Streptomycin 100 μg/ml and Trimethoprim (Tp) 100 μg/ml. Strains, plasmids and primers used in this study are listed in Tables 1, 2 and 3.

**Table 2:** Plasmids.

Plasmids	Description	Source
pDM4	Suicide vector (Cm^r^, R6K origin, *sacBR*)	Milton, *et al* 1996
pGEM**®-**T Easy	High copy number cloning vector (Amp^r^)	Promega
pRK2013	Conjugation, helper strain (Km^r^)	Lab stock
pSCrhaB3	Vector containing Tp^r^ cassette	Dr. C. Hemsley
pBHR4-groS-RFP	Vector containing Cm^r^ cassette	Dr. C. Hemsley
pSRC6a	pGEM®-T Easy containing 106-2A *icmF*2 left flanking region	This Study
pSRC7a	pGEM®-T Easy containing 106-2A *icmF*2 right flanking region	This Study
pSRC8a	pGEM®-T Easy containing 106-2A *icmF1* left flanking region	This Study
pSRC9b	pGEM®-T Easy containing 106-2A *icmF1* right flanking region	This Study
pSRC10	pDM4 containing containing *Nde*I ligated106-2A *icmF*2 left flank and right flank	This Study
pSRC11	pDM4 containing containing *Nde*I ligated106-2A *icmF1* left flank and right flank	This Study

**Table 3:** Primer sequences.

Primer Name	Description	Primer Sequence 5’–3’
M13 Forward	Sequencing primer	TGTAAAACGACGGCCAGT
M13 Reverse	Sequencing primer	CAGGAAACAGCTATGAC
LFF 106-2A *icmF*1 *SmaI*	106-2A left flanking region IcmF forward	TGCCCGGGAGTGATTGGTCTGAGGCCATTG
LFR 106-2A *icmF*1 *NdeI*	106-2A left flanking region IcmF reverse	TGCATATGTATCGGGTCTTGACGTAACTGG
RFF 106-2A *icmF*1 *NdeI*	106-2A right flanking region IcmF forward	TGCATATGTGGGCATTCTTTCGATTGTTAG
RFR 106-2A *icmF*1 *ApaI*	106-2A right flanking region IcmF reverse	TTGGGCCCCTCGGCACGATAAAGCTCTCTC
LFF 106-2A *icmF*2 *SmaI*	106-2A right flanking region IcmF2 forward	TGCCCGGGACCAGAGTGCGGATTATATTTC
LFR 106-2A *icmF*2 *NdeI*	106-2A left flanking region IcmF2 reverse	TGCATATGAATAACCAACAAACGGTTAAAG
RFF 106-2A *icmF*2 *NdeI*	106-2A right flanking region IcmF2 forward	TACATATGGCAAGGCCGCAATCTCGCAAAG
RFR 106-2A I *icmF*2 *ApaI*	106-2A right flanking region IcmF2 reverse	TCGGGCCCTGCCAATTTGAGGTAAACCATC
CmpDM4_F	first crossover integrant primer forward	ATGGAGAAAAAAATCACTGGATATACCACC
CmpDM4_R	first crossover integrant primer reverse	TTACGCCCCGCCCTGCCACTCATCGCAGTA
LFF_ *icmF1*_MutScr	Second cross over confirmation primer	GGCGGAAAGTGGAACAAAGC
RFR_ *icmF1*_MutScr	Second cross over confirmation primer	TACTCGGTTTGCTGGTACTC
LFF_ *icmF*2_MutScr	Second cross over confirmation primer	CGGCTGTGTTAGTCAGTGTG
RFR_ *icmF*2_MutScr	Second cross over confirmation primer	CTCATTCGATCACCGTTACC

### Identification of putative T6SS(s) in *V*. *vulnificus* 106-2A

WGS of *V*. *vulnificus* isolates was carried out by the Exeter Sequencing Service, University of Exeter, Exeter, UK. *De-novo* assembly of raw reads was performed using the A5-piepeline [36] followed by annotation of contigs using the RAST server [37]. Mapping of the putative T6SS clusters identified using RAST were performed using Clone Manager Professional Suite 8. Prior to GenBank submission assembled scaffolds were screened for contaminants against the UniVec database. Genomic features were predicted and annotated using prokka software and after manual curation submitted through the NCBI wgs portal. The genomes are accessible through the NCBI database with the following accession numbers: *Vibrio vulnificus* S2-22 (LKUU00000000); *Vibrio vulnificus* 106-2A (LMTD00000000); *Vibrio vulnificus* ORL-1506 (LMXV00000000); *Vibrio vulnificus* NSV-5830 (LMXW00000000); *Vibrio vulnificus 99–796* (LMXX00000000); *Vibrio vulnificus* 99–743 (LMXY00000000); *Vibrio vulnificus* DAL-79087 (LMXZ00000000); *Vibrio vulnificus* DAL-79040 (LMYA00000000); *Vibrio vulnificus* ATL-9824 (LMYB00000000); *Vibrio vulnificus* S3-16 (LMTC00000000); *Vibrio fluvialis* NCTC11327 (LMTE00000000).

### Generation of *V*. *vulnificus* SRC1 and SRC2 mutants

SRC1 and SRC2 mutants of *V*. *vulnificus* 106-2A were produced using pDM4 [38]. Flanking regions upstream and downstream of the target gene were PCR amplified using the primers described in Table 3. Correctly sequenced flanking regions were digested, purified and ligated into pDM4, resulting in pSRC10 and pSRC11, described in Table 2. pSRC10 and pSRC11 were subsequently transformed into *E*.*coli* DH5α and chromosomally introduced to *V*. *vulnificus* 106-2A using the conjugal donor pRK2013 and plated onto Cholera Medium TCBS (Oxoid) supplemented with choloramphenicol at 10 μg/ml. Meridiploid strains were confirmed using LFF or RFR flanking primers with cmpDM4_F or cmpDM4_R primers. Chromosomally confirmed first cross-over integrants were then plated onto LB supplemented with 10% sucrose (Sigma) incubated at 24°C to exercise the plasmid in a second cross-over event replacing the wildtype gene with the mutated gene. Mutants were confirmed using MutScr primers detailed in Table 3, DNA sequencing and WGS.

### Generation of antibiotic resistant *Vibrio* strains

Antibiotic resistant strains were generated using tri-parental conjugal mating using the helper strain pRK2013 and plated onto TCBS agar containing appropriate antibiotic.

### Hcp secretion analysis

Overnight *V*. *vulnificus* cultures were back-diluted to an OD_590_ 0.03 in 25 ml volumes and grown to mid-log OD_590_ 1.5. Cellular fractions were normalised to OD_590_ 1 in 1ml LB, centrifuged at 2000 rpm for 3 min, supernatant removed and pellets re-suspended in 50 μl bug buster, 25 μl PBS and 25 μl 4x loading buffer and heated at 100°C for 10min. Supernatants were filter sterilised using a 0.2 μm filter and proteins precipitated with 100% TCA on ice for 45mins and centrifuged at 20,000 g for 5mins at 4°C. Pellets were washed with acetone, heat dried at 95°C and re-suspended in sterile PBS. Precipitated proteins and cell pellets were quantified using the BCA assay (Thermo Fisher) and equal protein loadings were subjected to SDS-PAGE and Western blotting using primary Hcp-1 antibody [17] (1:500) and secondary anti-rabbit antibody. All were performed in conjunction with the strain SRC1 as a lysis control. Blots were visualised using Odyssey CLx Infared imaging system.

### Co-culture assays using TCBS enumeration plates

Overnight cultures of *Vibrio* strains were adjusted to OD_590_ 0.03 and grown to OD_590_ ~1.00, cultures were then re-adjusted to OD_590_ 0.8. Attacker and prey strains were then mixed at a ratio of 3 mls: 1 mls respectively. 200 ml of these mixed cultures were then incubated at 30°C for 5 hours on LB plates to allow contact dependent-killing in triplicate. Following incubation, co-cultures were harvested from the plates and re-suspended in 1 ml of sterile PBS for enumeration. 10-fold serial dilutions of these suspensions were prepared and 10 **μ**l of each dilution was spotted onto TCBS agar plates in triplicate. These enumeration plates were then incubated at 37°C overnight. The average cfu/ml was recorded for each triplicate dilution.

### Co-culture assays using selective antibiotic enumeration plates

Assays were performed as described above with the following modifications: prey and attacker strains were grown overnight in LB containing chloramphenicol (10mg/ml) and trimethoprim (100 mg/ml) respectively. Prior to mixing of the prey and attacker strains, overnight cultures were centrifuged and re-suspended in fresh LB media containing no antibiotic to prevent killing due to the antibiotics. Co-cultures using *V*. *fluvialis* NCTC 11327 as prey were incubated on LB plates at 30°C for five hours and co-cultures using *V*. *vulnificus* MO6-24/O as prey were incubated on LB plates at 30°C for two hours. Following incubation to determine T6SS dependent killing, co-cultures were harvested from the plates and re-suspended in 1 ml of sterile PBS for enumeration. 10-fold serial dilutions of these suspensions were prepared and 10 **μ**l of each dilution was spotted in triplicate onto LB agar plates that contained trimethoprim and onto another set of LB plates containing chloramphenicol. These enumeration plates were then incubated at 37°C overnight. The average cfu/ml was recorded for each triplicate dilution.

### *G*. *mellonella* infection assay

*G*. *mellonella* weighing between 2.0 and 3.0 g were injected in the upper most right proleg with *V*. *vulnificus* cells that had been grown overnight at 37°C in LB using a 25 μl Hamilton Microlitre™ syringe 800 series with removable needle (Sigma). Overnight bacterial cultures were adjusted to ~ 1x10^7^ cfu/ ml (equating to ~ 1x10^5^ cfu/10 μl). Enuermation of the infective dose was also performed to ensure the correct bacterial input. Infected larvae were then incubated at 37°C and monitored for signs of infection, death was recorded when larvae failed to respond to touch.

## Supporting Information

S1 Fig Representative gene maps of the T6SS2 of *V*. *vulnificus* isolates. Boxed maps are those of previously sequenced reference strains.
(PDF)

S2 Fig Representative gene maps of the T6SS1 of *V*. *vulnificus* isolates. (PDF)

S3 Fig Schematic demonstrating the deletion of *icmF1* from *V*. *vulnificus* 106-2A. (PDF)
