# Supplementary figures and images for: Vibrio vulnificus Type 6 Secretion System 1 Contains Anti-Bacterial Properties
Source: PLoS One. 2016 Oct 31;11(10):e0165500. doi: 10.1371/journal.pone.0165500 (PMC5087951; doi:10.1371/journal.pone.0165500)

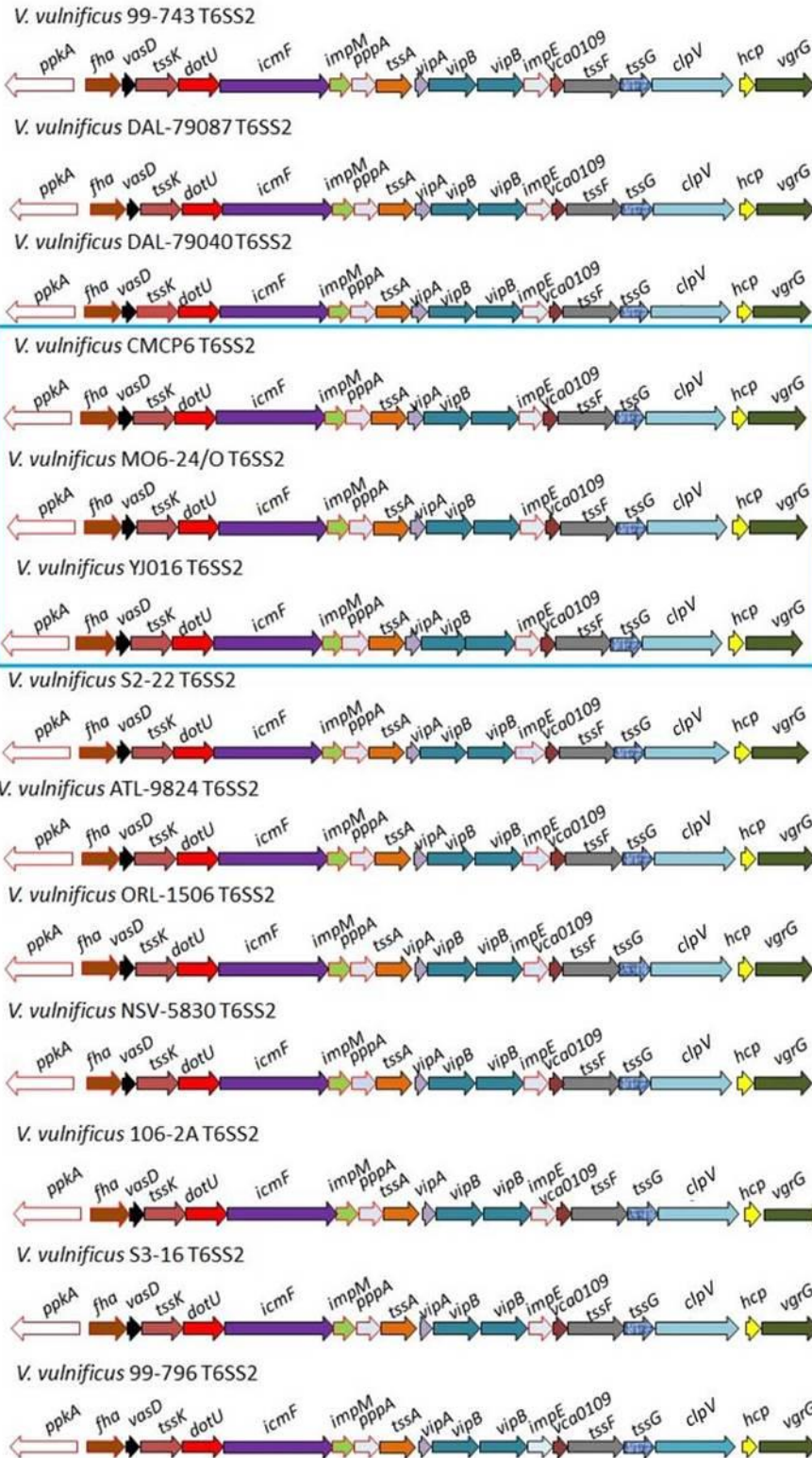

Supplement: S1 Fig — Boxed maps are those of previously sequenced reference strains. (PDF) [file pone.0165500.s001.pdf]

*V. vulnificus* 106-2A T6SS1

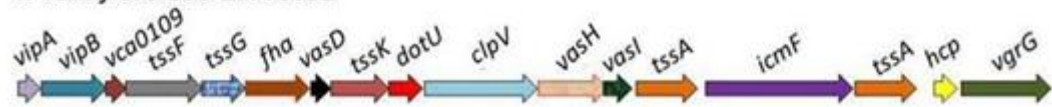

*V. vulnificus* S3-16 T6SS1

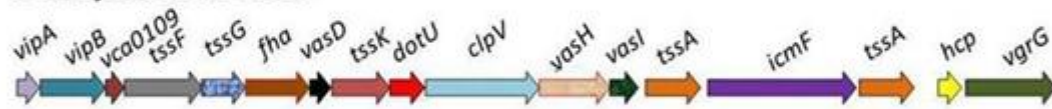

*V. vulnificus* 99-796 T6SS1

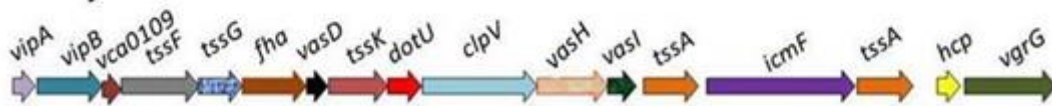

Supplement: S2 Fig — (PDF) [file pone.0165500.s002.pdf]

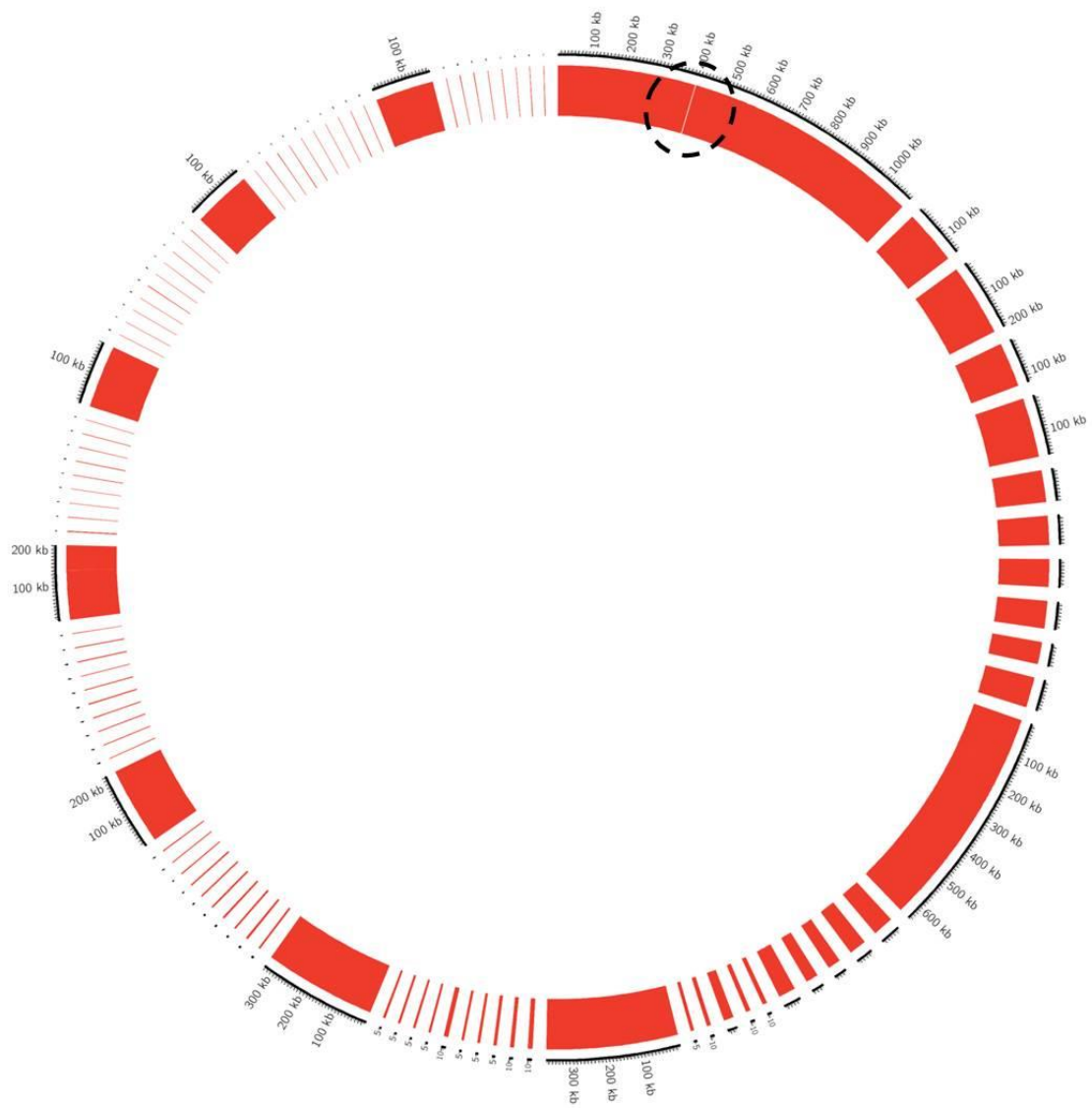

Supplement: S3 Fig — (PDF) [file pone.0165500.s003.pdf]
